# Advances in Pathogenesis and Therapeutics of Hepatobiliary Diseases II

**DOI:** 10.3390/biomedicines13040904

**Published:** 2025-04-08

**Authors:** Jing-Hua Wang

**Affiliations:** Institute of Oriental Medicine, Dongguk University, 32 Dongguk-ro, Goyang 10326, Republic of Korea; wjhdon@dongguk.edu; Tel.: +82-31-961-8418

Hepatobiliary diseases, including liver fibrosis, cirrhosis, hepatocellular carcinoma (HCC), and cholestatic liver disorders, pose significant global health challenges due to their complex pathogenesis and limited treatment options [1]. Recent advances have deepened our understanding of key molecular mechanisms, such as metabolic dysfunction, immune responses, and gut–liver axis interactions, while novel therapeutic strategies, including targeted therapies, immunotherapy, and natural compounds, show significant potential in improving patient outcomes [2,3,4,5].

This Special Issue, “Advances in Pathogenesis and Therapeutics of Hepatobiliary Diseases II”, showcases cutting-edge research articles and reviews that further explore these advances. Covering a wide range of topics, from molecular mechanisms and diagnostic innovations to novel therapeutic and preventive strategies, this Special Issue features seven articles from eight countries, including five original research papers and two reviews. These studies address major hepatobiliary conditions such as non-alcoholic fatty liver disease (NAFLD), primary biliary cholangitis (PBC), hepatitis B/C, and hepatocellular carcinoma (HCC). Detailed summaries are presented below. We believe that this collection provides valuable insights and will contribute to the ongoing advancement of hepatobiliary disease research and clinical management.

Chayanupatkul et al. investigated the effects of single- and mixed-strain probiotics on non-alcoholic steatohepatitis (NASH) in a rat model induced by a high-fat, high-fructose diet [6]. Both probiotic treatments significantly reduced hepatic fat accumulation and inflammation, improved liver histology, and altered the gut microbiota composition, particularly increasing beneficial bacteria such as Lactobacillus and reducing harmful ones such as Akkermansia. Probiotic supplementation also reduced the expression of inflammatory markers TLR4 and CD14 and lowered serum TNF-α and IL-6 levels. Notably, this was the first study to evaluate the combined effects of *L. rhamnosus* L34 and *L. paracasei* B13 in NASH, showing potential therapeutic benefit through gut–liver axis modulation.

In addition, Palmiotti et al. investigated the therapeutic effects of the bile acid sequestrant colesevelam in Cyp2c70−/− mice, which have a human-like bile acid composition and develop liver fibrosis [7]. Colesevelam reduced bile acid hydrophobicity, normalized liver damage markers, and lowered fibrogenesis-related gene expression, thereby improving liver pathology without affecting insulin sensitivity. Notably, colesevelam shifted the bile acid composition toward less cytotoxic and more hydrophilic species by promoting 12α-hydroxylated bile acids. This study demonstrates that bile acid sequestration can alleviate liver damage in a humanized mouse model, offering insights into potential treatments for cholestatic liver diseases.

Moreover, Legaz et al. examined the role of killer cell immunoglobulin-like receptor (KIR) and human leukocyte antigen (HLA)-C genetic profiles in the development of ascites in male alcoholic cirrhosis (AC) patients. The results showed that reduced frequencies of KIR2DL2 and KIR3DL1 genes may predispose AC patients to ascites, while the KIR2DS2 and KIR2DS5 genes could be involved in disease susceptibility. Additionally, specific HLA-C genotypes, such as C1C2 and C2C2, were identified as potential protective or risk factors. This study identified KIR/HLA gene profiles as potential biomarkers for predicting ascites risk in AC patients, providing new insights into personalized risk assessment and management strategies.

Etzel et al. systematically evaluated different interventional strategies in transarterial radioembolization (TARE) therapy to prevent extrahepatic microsphere deposition, a critical risk factor for severe complications [8]. By analyzing 398 TARE procedures, the authors compared three approaches: a single treatment position (TP) with and without interventional occlusion (IO), and multiple TPs without IO. The results demonstrated that applying microspheres from multiple TPs significantly reduced the risk of extrahepatic deposition without increasing angiographic complications. Notably, 50.9% of procedures requiring IO could have safely avoided by using multiple TPs. The study proposes multiple TPs as a safer and more flexible alternative to traditional prophylactic vessel occlusion. This approach optimizes microsphere delivery, minimizes risks, and enhances treatment precision, providing a new perspective on interventional strategy selection for TARE. Drescher et al. evaluated the clinical outcomes of transarterial radioembolization (TARE) using holmium-166 microspheres (166Ho-TARE) in patients with primary and secondary liver cancers [9]. Among 20 patients, 166Ho-TARE achieved good local tumor control and treatment-free intervals and served as a bridging therapy for liver transplantation or curative surgery in some cases. The treatment was feasible and well tolerated and did not prevent subsequent therapies. This demonstrated the clinical utility of 166Ho-TARE as a flexible and effective option within multidisciplinary oncology, with potential advantages such as personalized dosimetry and imaging capabilities for improved treatment planning.

The first review in this Special Issue focuses on the complex role of gut microbiota (GM) dysbiosis in hepatocellular carcinoma (HCC) pathogenesis and treatment, particularly its impact on immunity, bile acid metabolism, and responses to immune checkpoint inhibitors (ICIs) [10]. It identifies GM as a potential biomarker and therapeutic target to enhance ICI efficacy, addressing the current lack of reliable response predictors. This review provides a novel perspective on the integration of microbiota analysis into precision HCC treatment strategies. The second review summarizes recent advances (2018–2023) in the role of probiotics in treating hepatobiliary diseases by modulating the gut microbiota and the gut–liver axis [11]. It highlights how specific probiotic strains alleviate liver conditions such as NAFLD, NASH, ALD, autoimmune hepatitis, and cholestatic diseases through mechanisms such as improving intestinal barrier function, regulating bile acids, and reducing inflammation. A major innovation involves the integration of bioinformatics to explore probiotic mechanisms and potential new therapeutic strains, paving the way for personalized probiotic-based therapies. This review also emphasizes the need for further research on the safety, viability, and clinical dosage of probiotics.

Collectively, this collection of articles offers valuable insights that can advance the diagnosis, prevention, and treatment of hepatobiliary diseases, ultimately contributing to improved patient outcomes and enhanced public health.
